# Approved Nanomedicine against Diseases

**DOI:** 10.3390/pharmaceutics15030774

**Published:** 2023-02-26

**Authors:** Yuanchao Jia, Yuxin Jiang, Yonglong He, Wanting Zhang, Jiahui Zou, Kosheli Thapa Magar, Hamza Boucetta, Chao Teng, Wei He

**Affiliations:** 1 Nanjing Vtrying Pharmatech Co., Ltd., Nanjing 211122, China; 2 School of Pharmacy, China Pharmaceutical University, Nanjing 211198, China; 3 Shanghai Skin Disease Hospital, Tongji University School of Medicine, Shanghai 200443, China

**Keywords:** nanomedicine, liposomes, nanocrystal, polymeric nanoparticles, cancer, cardiovascular disease, infection

## Abstract

Nanomedicine is a branch of medicine using nanotechnology to prevent and treat diseases. Nanotechnology represents one of the most effective approaches in elevating a drug‘s treatment efficacy and reducing toxicity by improving drug solubility, altering biodistribution, and controlling the release. The development of nanotechnology and materials has brought a profound revolution to medicine, significantly affecting the treatment of various major diseases such as cancer, injection, and cardiovascular diseases. Nanomedicine has experienced explosive growth in the past few years. Although the clinical transition of nanomedicine is not very satisfactory, traditional drugs still occupy a dominant position in formulation development, but increasingly active drugs have adopted nanoscale forms to limit side effects and improve efficacy. The review summarized the approved nanomedicine, its indications, and the properties of commonly used nanocarriers and nanotechnology.

## 1. Introduction

Nanoparticles are particles with a size range of 1–100 nm or products with a particle size outside this range but whose preparation characteristics are generally related to the particle size [1,2]. Nanomedicine uses nanoparticles such as polymeric micelles, liposomes, and lipid nanoparticles in living organisms for disease prevention and treatment [3]. The clinical use of traditional drugs is always limited due to their water-solubility, stability, poor pharmacokinetics, low bioavailability, low targetability, toxicity, etc [4]. Nanomedicine emerged to overcome these problems and has made significant progress. Nanomedicine improves the pharmacokinetic behavior of drugs and reduces toxicity by improving drug solubility, altering biodistribution, and controlling the release [5]. The large surface area of nanoparticles offers an enhanced interaction with cells and effectively increases the intracellular drug concentration [6]. The surface modification also allows increased drug accumulation in the lesions and penetrability [7]. In addition, through codelivery, the advantages of synergistic therapy can be improved and compromise drug resistance [8]. Nanomedicine has obtained breakthroughs in cancer treatment, diagnosis, and gene delivery [9,10,11,12,13], as more than 90 nanoformulations were marketed [14,15,16].

The development progress of nanomedicine can be divided into three stages. The first stage is a primary research stage that lasted for 30 years, from the discovery of liposome structure in 1964 to the first approved nanomedicine by the Food and Drug Administration (FDA) based on liposomal doxorubicin (DOX) delivery system, Doxil^®^ in 1995 [17]. The second stage lasted from 1995 to 2007 and mainly involved clinical validation and commercialization. The third is the stage, including the rapid nanomedicine development, in which various innovative nanomedicine appeared. Although nanomedicine had higher treatment efficacy than traditional drugs, it could not balance pathological-tissue regulation and normal-organ protection. In order to solve this problem, smart nanomedicines with the reaction to endogenous and exogenous stimuli have been developed to ensure more precise disease treatment and minimize the non-specific and related toxicity. Several of them have entered clinical trials [18]. Consequently, improved delivery using nanoscaled drug delivery systems (NDDSs) to the lesions is the hottest research area in nanomedicine, accounting for half of the articles published in the past 20 years. The NDDSs, with certain therapeutic effects such as nanocrystals of insoluble drugs, deliver drugs to specific tissues of the body. NDDSs developed rapidly over the few years. For instance, the research articles regarding NDDSs increased over ten-fold from 2000 to 2019 [19]. Commonly used NDDS include nanocrystals, nanoemulsions, liposomes and lipid nanoparticles, polymers, protein-based nanoparticles, etc. [20]. So far, more than 90 NDDSs have been marketed. NDDSs are attracting worldwide attention due to their excellent therapeutic effects and are becoming one of the top five promising technologies, according to the Forbes report [21]. Several reviews summarized different types of nanomedicine against a specific disease, and cancer accounts for the majority [22,23,24]. Other diseases, such as infection and cardiovascular disease, are increasingly threatening human health. This review focuses on NDDS-based medicine against major diseases that threaten human life. We introduced the marketed nanomedicine and its main characteristics. Then, we analyzed the approved formulations and outlooked the field.

## 2. Liposomes and Lipid Nanoparticles (LNPs)

Over the past few years, liposomes and LNPs have been through a long and tortuous road from the concept to the mainstream status of lipid carriers in NDDSs. Since the first liposomes entered clinical trials in 1985, over 20 liposomes and LNPs have been approved for marketing (Table 1). The success of liposomal drugs further stimulated extensive clinical research on lipid-related nanoparticles. They have been clinically explored for disease treatment by vaccine and gene-drug delivery.

### 2.1. Liposomes

Since Alec Bangham first revealed that phospholipids could form a closed bilayer structure in an aqueous system [25], liposomes, as a closed bilayer phospholipid system, demonstrated the ability to load hydrophilic and hydrophobic drugs in a bilayer structure and aqueous cores. Liposomes possess numerous advantages, such as high biocompatibility, low immunogenicity, and the ability to easily change the size, charge, and surface properties by modifying the prescription or preparation methods, increasing efficiency and decreasing toxicity [25]. Additionally, modifying the liposomal membrane allows prolonged blood circulation time and increased lesion accumulation of the drug [26]. So far, the liposomes have developed into mature drug carriers. The use of the extrusion technique for homogeneous size and the PEGylated approach for long-circulating facilitated liposome translation [27]. The emergency of enhanced permeability and retention (EPR) effect first described in 1986 in solid tumors further derived the translation, allowing increased accumulation of nanoparticles in tumor tissue due to the increased vascular permeability. Liposomes are the first nanomedicine that is translated into clinical use. For example, AmBisome^®^ and Doxil^®^ demonstrate considerable clinical success, with annual sales in the hundreds of millions of dollars [28]. The section displayed the approved liposomal formulations and their clinical use.

#### 2.1.1. Liposomes against Cancer

Cancer is amongst the most severe public health problems menacing the world. Approximately 1.92 million new cancer cases and 0.61 million new cancer deaths will be estimated in developed countries in 2022 [29]. Due to lacking selectivity, traditional small-molecule chemotherapeutic drugs often cause severe damage to normal tissues and organs, such as bone marrow and gastrointestinal tract, while killing tumor cells [30,31]. In recent years, nanomedicines have been extensively used in antitumor therapy as increasing research focuses on efficient and low-toxic NDDSs. Among them, liposomes are a hot spot with various antitumor liposomes being marketed.

DOX liposomes are the most widely studied antitumor liposomes. DOX has strong antitumor effects against several tumors by inhibiting DNA and RNA synthesis in tumor cells [32]. DOX has potential mutagenic and carcinogenic activity and adverse effects, including cardiotoxicity and bone marrow inhibition. Liposomes enable the passive targeting of drugs to tumor tissues through the EPR effect in solid tumors and reduce drug toxicity. The first marketed DOX liposome was Doxil^®^ developed by Sequus in the United States (US), mainly for treating recurrent ovarian cancer and human immunodeficiency virus (HIV)-induced Kaposi’s sarcoma (KS) [33,34]. Doxil^®^ uses STEALTH^®^ technology to encapsulate DOX in a polyethylene glycol (PEG)-modified stealth liposome composed of synthetic phospholipids and has prolonged blood circulation, with a prescription composition molar ratio of HSPC:CHOL: DSPE-mPEG2000 = 56:39:5 and a particle size of about 100 nm [35]. Using synthetic phospholipids as carriers offers enhanced stability, accompanied by controlled release due to PEG modification, thus significantly reducing the cardiotoxicity caused by DOX [36]. Nevertheless, PEG modification has also been associated with concentration-dependent toxicity. When high doses of PEG-modified liposomes are administrated, some of the leaked drugs may lead to “hand and foot syndrome”, characterized by numbness of hands and feet [37,38]. Therefore, Myocet^®^, a non-PEGylated modified DOX liposome developed by Elan Pharmaceuticals, was developed in Europe, in which DOX is present in the form of citrate. Compared to Doxil^®^, Myocet^®^ has a more straightforward prescription composition with a 55:45 molar EPC to CHOL, a drug-lipid ratio of 0.27, and a 150–250 nm diameter. Myocet^®^ did not show apparent toxicity toward the skin in treating metastatic breast cancer. Nevertheless, due to the lack of PEG modification, Myocet^®^ is rapidly phagocytosed by macrophages in vivo, showing no advantage over PEGylated liposomes in terms of half-life and tumor targeting [39].

In addition to enhancing drug retention at the tumor site via the EPR effect, liposomes can simultaneously deliver different antitumor drugs for efficacy improvement [40]. In 2017, Vyxeos^®^ (CPX-351), characterized by the co-loading of cytarabine and daunorubicin, was developed for treating therapy-related acute myeloid leukemia (t-AML) and AML with myelodysplasia-related changes [41,42]. Sustained synergy-treatment efficacy and reduced side effects were obtained by CombiPlex^®^ technology (cytarabine: daunorubicin = 5:1) [42,43].

Currently, most of the marketed antitumor liposomes carry small molecule drugs. Besides the mentioned liposomes above, there are other marketed small molecule antitumor liposomes, such as Marqibo^®^, a vincristine liposome against acute lymphoblastic leukemia (ALL) [44], Depocyt^®^, a cytarabine liposome for neoplastic meningitis (NM) [45], and Onivyde™, an irinotecan liposome for advanced pancreatic cancer [46]. Additionally, there are antitumor liposomes encapsulating macromolecular drugs on the market. Mepact^®^ is a mifamurtide (MFT) liposome developed by IDM Pharma, an orphan drug for treating osteosarcoma. Mepact^®^ is a multilayer liposome with a particle size of 2.0 to 3.5 μm, containing the active ingredients cytosolic acyl tripeptide phosphatidylethanolamine (MTP-PE), palmitoyl oil-based phosphatidylcholine (POPC) and dioleoyl phosphatidylserine (OOPS) in a molar ratio of POPC: OOPS = 7:3 and MTP-PE: phospholipid = 1:250. This particle size facilitates monocyte/macrophage recognition, phagocytosis, and drug release, killing cancer cells by activating monocytes and macrophages [47].

#### 2.1.2. Liposomes against Infection

Besides cancer, infectious diseases also threaten human health and life. Viruses, bacteria, and fungi are the three most common groups of pathogenic microorganisms [48]. Anti-infective drugs can be divided into several types, including anti-viral drugs (adamantanamine, zidovudine, ribavirin, and interferon), anti-bacterial drugs (penicillin, macrolides, metronidazole), anti-fungal drugs (fluconazole, itraconazole, and voriconazole) and anti-parasitic drugs (albendazole and praziquantel), according to the different pathogens of infection [49].

Leishmaniasis is a zoonosis caused by intracellular protozoan parasites belonging to Leishmania. The World Health Organization (WHO) estimates 2–2.5 million cases of leishmaniasis each year [50]. Pentavalent antimonial is the first-line drug for treating visceral leishmaniasis, but its adverse events or toxic reactions happen frequently, and even ineffective reactions occur [51]. Amphotericin B, a polyene macrolide antibiotic that binds to sterols in cell membranes, is currently the most effective anti-leishmanial drug [52]. Due to its poor oral absorption, only dosing concentrations can be achieved by intravenous injection. Moreover, it is easy to bind with serum protein and accumulate in the liver, lung, spleen, and kidney, resulting in toxicity [53]. The application of liposomes significantly reduced the drug‘s toxicity [54]. AmBisome^®^ and Amphotec^®^ marketed in 1990 and 1996, respectively, are effective and well-tolerated to combat visceral leishmaniasis in immunocompetent patients and other severe fungal infections. AmBisome^®^ obtains fungicidal activity and inhibits fungal replication by penetrating the fungal cell wall, entering the cell, releasing the drug, and binding to the sterol component of the membrane (mainly ergosterol) [55]. Abelcet^®^ is composed of two lipids (dimyristoyl-phosphatidyl-choline and dimyristoyl-phosphatidylglycerol) in a 7:3 molar ratio. The formulation is effective in patients with invasive fungi who do not respond to or are intolerant to conventional anti-fungal therapy [56]. Subsequently, increasing liposomal products against infection were marketed with more administration routes, not limited to intravenous administration.

Inflexal^®^ is an inactivated subunit influenza vaccine with a virion adjuvant and was commercialized in 1997 [57]. It is tolerable, safe, and effective in all age groups in various pre-and post-marketing studies [58]. Launching the Mosquirix^®^ vaccine is an essential step in fighting against malaria, a parasitic infection whose symptoms range from moderate fever to nervous system disorders. Mosquirix^®^ was advocated by the WHO in 2021 for its inclusion in routine immunization schedules and existing malaria control measures, although the economic cost of the vaccine is high [59]. Both vaccines are administered by intramuscular injection. Launching Arikayce^®^ Kit represents an advance in the liposomal ultra-complex formulation. Arikayce^®^ Kit is a liposomal inhalation formulation administered using the eFlow nebulization system manufactured by PARI Pharma GmbH for treating non-tuberculous mycobacterial (NTM) lung disease, specifically NTM lung disease caused by Mycobacterium avium complex (MAC), the first and only therapy specifically designed to treat this unique lung disease in the US [60].

As penicillin was invented, the human‘s ability against bacteria, viruses, and fungi was significantly strengthened. More than 10 lipid-based products against infectious diseases were marketed. However, the incidence of infectious diseases worldwide is still growing, and the pathogens show a trend of diversification and complexity. Due to the widespread application of antimicrobial drugs, bacterial resistance to drugs occurs. Antibiotic Stewardship (ABS) is a crucial method to prevent the spread of drug-resistant pathogens and the emergence of multidrug resistance [61]. The measurement to prevent drug resistance includes implementing local guidelines, developing location-specific anti-infection lists, regular quarantine visits, practice-oriented in-house training activities, and minimizing antibiotic use in an outpatient setting. Liposomal formulations effectively perform the ABS and combat drug resistance by reducing doses and frequency. Nonetheless, there is still an urgent requirement to develop new anti-infective drugs. Virtual high-throughput screening and structure-based rational drug design have been established as powerful tools [62]. In addition, alternative non-antibiotic approaches must be actively sought, such as identifying new targets in pathogenic microbial cells (quorum sensing, riboswitches, transcriptional regulators, etc.) [63].

#### 2.1.3. Liposomes for Pain Relief and Other Disease Therapy

Liposomes are also being used in other treatment areas, such as pain management [64,65] and photodynamic therapy (PDT) [66]. Two liposomal products are used for clinical analgesia: the morphine liposome, DepoDur™, and the bupivacaine liposome, Exparel^®^. Both are produced using the slow-release multivesicular liposome preparation technology, Depofoam™. DepoDur™ is a liposome encapsulating morphine sulfate primarily used to relieve postoperative pain. DepoDur™ has an extended drug release time compared to regular morphine preparations, with analgesic effects lasting up to 48 h after a single dose, approximately twice as long as regular morphine preparations [67,68]. Exparel^®^ also has a long-lasting analgesic effect, achieving 72 h of pain relief after subcutaneous injection, whereas the pain suppression time of regular bupivacaine injection is only 7 h [69,70].

Liposomes can release drugs in vivo in different ways, including in response to pH alterations, temperature changes, or other conditions [71,72,73]. Visudyne^®^ is a combined PDT formulation of liposomes for treating age-related macular degeneration (AMD) and choroidal neovascularization (CNV) [74]. Visudyne^®^ irradiates the lesion with a non-thermogenic red-light source at a specific wavelength of 689 ± 3 nm, where a photochemical reaction forms singlet oxygen to produce local cytotoxic effects and generates reactive oxygen radicals, which damage local neovascular endothelial cells and help vascular closure [75]. The light-triggered release of the contents offers Visudyne^®^ photodynamic therapy with low toxicity and high selectivity.

Liposomes, one of the most successfully used nanomedicine, possess promising application prospects and numerous advantages. However, several liposome formulations failed in clinical trials because they did not reach the expected endpoints. The key to solving liposomes’ clinical translation is technical issues, such as scale-up synthesis, performance optimization, prediction, etc. In the future, we expect to see more liposome products successfully applied in clinical practice, and we desire that liposome’s clinical and commercial applications could stimulate and promote the clinical translation of other nanomedicines.

### 2.2. LNPs against Hereditary Transthyretin Amyloidosis (hATTR) and COVID-19 Infection

Liposomes are potent to encapsulate small-molecule drugs and improve their delivery; however, they are always modest in delivering biopharmaceuticals due to poor encapsulation efficacy and endosomal escape. LNPs are appearing to enhance the delivery of biologics with high-molecule weight and poor membrane penetrability and stability [76]. LNPs comprise ionizable lipids, phospholipids, cholesterol, and PEG-lipid conjugate. All lipid components have their specific functions [77,78]. The phospholipids (distearoyl phosphatidylcholine (DSPC), dipalmitoyl phosphatidylcholine (DPPC)) form the basic backbone of lipid bilayers; cholesterol is mainly used to regulate the rigidity and mobility of liposomes and to stabilize the structure of liposomes; ionizable lipids have pKa of 6–7 and contain amino groups such as G0-C14 and C12-200. The lipids are generally negatively charged while becoming positively charged in weak acid conditions and facilitate the lysosomal escape; other lipid components help reduce non-specific uptake [79,80,81].

**Figure 1:**
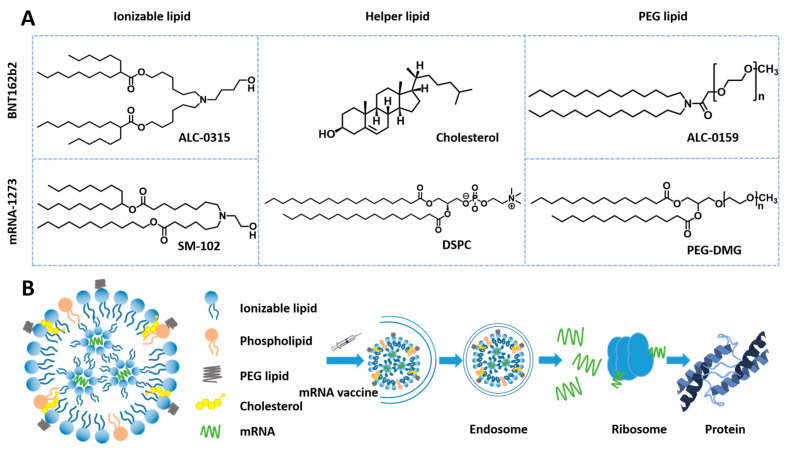
Construction of mRNA-1273 and BNT162b2 and active mechanism. (**A**) Composition and structure of the two mRNA vaccines. (**B**) Active mechanism of mRNA vaccine.

LNPs, as one of the most studied nanocarriers, undergo rapid technological evolution and get significant advances. For instance, the first siRNA drug, Onpattro™ (a new small interfering RNA (siRNA) LNP), was launched in 2018 for treating hATTR polyneuropathy, signifying a milestone advancement in LNP technology [82]. The key to Onpattro™ success is the development of ionizable cationic lipids. Upon arrival at endosomes, the ionizable lipids of LNPs undergo charge flipping to positive charge in an acidic environment, leading to endosomal escape and significantly improving the efficiency of in vivo delivery of unstable nucleic acid drugs [83]. Nucleic acid therapies, such as mRNA, are evolving into precise medicines that can manipulate specific genes. However, their large size and vulnerability greatly limit their clinical use [84]. Under the current impact of COVID-19 [85,86], LNPs, as a critical component of the messenger RNA (mRNA) vaccine, play an essential role in protecting and delivering mRNA [87]. The two approved mRNA vaccines, mRNA-1273 and BNT162b2 used LNPs as carriers and showed significant effectiveness for infection prevention. The effectiveness of the mRNA-1273 and BNT162b2 is 94.1% and 95.0%, respectively. The robust efficacy is closely linked to the structure and active mechanism (Figure 1). During the endosomal escape, ionizable lipids protonate [88] and interact with the endosomal membrane to form destructive non-bilayer structures when pH is below the pKa of ionizable lipids, ultimately leading to the release of mRNA [89]. The two COVID-19 mRNA vaccines are Comirnaty expressing the S protein from Moderna (Cambridge, MA, USA) [90], and Spikevax encoding the RBD protein from BioNTech (Mainz, Germany) [87,91]. Nonetheless, the mRNA COVID-19 vaccines from Moderna and BioNTech/Pfizer (New York, NY, USA) must be kept between −15 and −25 °C and between −60 and −90 °C, compared with 2–8 °C of other vaccines. These temperature-demanding storage conditions make vaccine transportation and distribution costs dramatically higher.

Overall, liposomes account for most of the nanomedicines marketed to date. The two nanocarriers demonstrate advantages over other NDDSs, such as polymer micelles and dendrimers, including higher payload ability, enhanced stability, drug protection, biocompatibility, and more straightforward modification and industrialization. Moreover, liposomes have two drug-loading sites and demonstrate unique advantages for co-delivery. For instance, liposomes allowed the approval of co-delivery nanomedicine, Vyxeos^®^, to treat AML. However, most products are dosed by intravenous route (most vaccines are intramuscular injection) [92]. Gastrointestinal degradation of the carrier can reduce the bioavailability of the drug, so oral administration is generally not appropriate for lipid-carrier products [92].

**Table 1:** Marketed liposome- and LNP products.

Trade Name	Approval Year	Drug Agent	Company	Clinical Applications	Agency	Administration Route	Ref.
Ambisome^®^	1990	Amphotericin B	Gilead Sciences (Foster, CA, USA)	Fungal infection/Anti-leishmanial	EMA	intravenous	[55,93,94]
Epaxal^®^	1993	Inactivated hepatitis A virus (strain RGSB)	Crucell Berna Biotech (Berne, Switzerland)	Hepatitis A	EMA	intramuscular	[95,96]
Abelcet^®^	1995	Amphotericin B	Sigma-Tau Pharmaceutical Inc. (Gaithersburg, MD, USA)	Invasive severe fungal infections	FDA	intravenous	[56]
Doxil^®^/Caelyx^®^	1995/1996	Doxorubicin	Sequus Pharmaceuticals (Santa Clara County, CA, USA)	Ovarian cancer and KS	FDA/EMA	intravenous	[33,34,97]
Amphotec^®^	1996	Amphotericin B	Ben Venue Laboratories (Bedford, OH, USA)	Severe fungal infections	FDA	intravenous	[98]
DaunoXome^®^	1996	Daunorubicin	NeXstar Pharmaceuticals (Foster, CA, USA)	KS infected with HIV	FDA	intravenous	[99]
Inflexal^®^ V	1997	Inactivated hemagglutinin of Influenza virus strains A and B	Crucell Berna Biotech (Berne, Switzerland)	Influenza	EMA	intramuscular	[57]
Depocyt^®^	1999	Cytarabine	Skye Pharm Inc. (San Diego, CA, USA)	Neoplastic meningitis	FDA	spinal	[100,101]
Visudyne^®^	2000	Verteporfin	Novartis AG (Basel, Switzerland)	Choroidal neovascularization	FDA	intravenous	[75]
Myocet^®^	2001	Doxorubicin	IDM Pharma (Irvine, CA, USA)	Combination therapy with cyclophosphamide in metastatic breast cancer	EMA	intravenous	[102]
Lipusu^®^	2003	Paclitaxel	Luye Pharma (Nanjing, China)	Ovarian cancer	NMPA	intravenously guttae	[103]
DepoDur™	2004	Morphine Sulfate	SkyPharm Inc. (San Diego, CA, USA)	Pain management	FDA	Epidural	[68]
Mepact^®^	2009	Mifamurtide	Elan Pharmaceuticals (San Diego, CA, USA)	Non-metastatic osteosarcoma	EMA	intravenous	[47,104]
Exparel^®^	2011	Bupivacaine	Pacira BioSciences (San Diego, CA, USA)	Pain management	FDA	intravenous	[105]
Marqibo^®^	2012	Vincristine	Talon Therapeutics (San Francisco, CA, USA)	ALL	FDA	intravenous	[44]
Onivyde™	2015	Irinotecan	Merrimack Pharmaceuticals (Cambridge, UK)	Metastatic pancreatic cancer	FDA	intravenous	[46,106]
Vyxeos^®^	2017	Daunorubicin and Cytarabine	Jazz Pharmaceuticals (San Francisco, CA, USA)	AML-MRC and t-AML	FDA	intravenous	[41,42]
Shingrix^®^	2017	Recombinant VZV glycoprotein E	Glaxo Smith Kline (Middlesex, UK)	Against shingles and post-herpetic neuralgia	FDA	intramuscular	[107,108]
Onpattro™	2018	siRNA	Alnylam (Cambridge, MA, USA)	Polyneuropathy caused by hATTR	FDA	intravenous	[82,109,110]
Arikayce^®^ Kit	2018	Amikacin	Insmed (Glen Allen, VA, USA)	NTM lung disease caused by MAC	FDA	inhalation administration	[111,112]
Mosquirix^®^	2021	Recombinant CSP	Glaxo Smith Kline (Middlesex, UK)	Malaria	EMA	intramuscular	[59]
Comirnaty^®^	2021	BNT162b2	Pfizer (New York, NY, USA) and BioNTech (Mainz, Germany)	COVID-19	FDA	intramuscular	[113]
mRNA-1273	2021	mRNA-1273	Moderna (Cambridge, MA, USA)	COVID-19	FDA	intramuscular	[114]

FDA: Food and Drug Administration; EMA: European Medicines Agency; NMPA: National Medical Products Administration.

## 3. Drug Nanocrystals (NCs)

Over the last two decades, over 15 NCs were approved for clinical use (Table 2). Compared to lipid-based and polymeric nanocarriers, NCs outstand delivering hydrophobic drugs through multiple administration routes and excel in many different areas, such as high drug-loading capacity, long-term stability, enhanced release, barrier penetration, and easily scalable techniques [115]. Significantly, NCs are the most promising nanomedicine for long-lasting activity against diseases due to their extremely high drug-loading capacity. Mostly, two important techniques have been used for NCs fabrication, bottom-up and top-down approaches [116,117,118]. In this part, an elaboration of approved clinical trials and some encouraging preclinical NCs‘ characteristics and features will be found, catching a glimpse, particularly for those used in cancer, cardiovascular diseases (CVDs), and infections.

### 3.1. NCs against CVDs

CVDs are one of the major causes of morbidity and mortality worldwide, which include a diverse range of heart and circulatory system dysfunctions such as coronary artery diseases, stroke, peripheral arterial diseases, myocardial infarction (MI), and aortic diseases [10,119,120,121,122]. Increased blood cholesterol levels are a risk factor, biomarker, and prediction of CVDs since cholesterol accumulates in blood vessel walls and can restrict or obstruct blood flow and oxygen delivery [121,123]. The first approved NCs product employed to prevent the development of atherosclerosis and the plaques on the inner wall of arteries that cause strokes and heart attacks were Tricor^®^, fenofibrate NCs back on 5 November 2004 [124]. Fenofibrate is a water-insoluble drug that belongs to class II in the Biopharmaceutical Classification System (BCS) and is clinically used to reduce the plasma level of low-density lipoproteins (LDL) and cholesterol in patients with hypercholesterolemia [125]. To improve the oral bioavailability of fenofibrate, Abbott Lab developed Tricor^®^ utilizing the pearl mill technology, producing particles of below 30 nm in size; compared to micronized fenofibrate, it showed higher drug solubility and enhanced oral bioavailability by 9% without affecting its effect in a fed or fasted state [126]. The fenofibrate NCs were encapsulated in different water-soluble polymers, including polyvinylpyrrolidone, polyvinyl alcohol (PVA), and hydroxypropyl methylcellulose (HPMC) [127]. Gite et al. fabricated PVA containing surface-engineered fenofibrate NCs via the media milling method, and these formulations showed a quick and complete dissolution within 30 min compared to Tricor^®^. In vivo pharmacokinetics study demonstrated that the novel fenofibrate formulations had a remarkably improved bioavailability and faster onset of action, with a 51.46% shorter T_max_, 82.63% higher C_max_, and 69.34% higher AUC_0–24 h_, respectively [128]. Other techniques have also been used to prepare fenofibrate NCs, such as evaporation-assisted antisolvent interaction using polymers HPMC, polyvinylpyridine (PVP) and PVA, high-pressure homogenization (HPH) method using poloxamer 188 (P188) and Tween 80. Both formulations showed accelerated solubility and dissolution, vastly enhancing drug absorption [127,129]. Another advanced fenofibrate NCs product (Triglide^®^), approved on 7 May 2005, was formulated via the HPH method, and each tablet of Triglide^®^ contains active fenofibrate (50 mg or 160 mg) and other inactive agents (crospovidone, lactose monohydrate, mannitol, maltodextrin, carboxymethylcellulose sodium, egg lecithin, croscarmellose sodium, sodium lauryl sulfate, colloidal silicon dioxide, magnesium stearate, and monobasic sodium phosphate). Triglide^®^, a 160-mg tablet, exhibits a 32% higher absorption rate than the 200 mg micronized fenofibrate capsule under low-fat-fed conditions [130,131].

### 3.2. NCs against Infection

Anti-infective agents, including anti-bacterial, anti-fungal, anti-viral, and anti-parasitic agents, have been employed to treat different infectious diseases such as severe acute respiratory infections (coronaviruses: SARS-CoV-2/COVID, SARS-CoV, and MER), HIV infection caused by various pathogenic agents, i.e., viruses, bacteria, fungus, and parasites [132,133]. The first NCs-based product to receive US FDA approval for the treatment of fungal infection was orally administered Gris-PEG, which was manufactured by Recro Gainesville LLC (Gainesville, FL, USA) in 1998 and approved for oral administration of the anti-fungal drug griseofulvin to treat ringworm infection. Each Gris-PEG^®^ tablet (Ultra-microsize: 10–30 μm) was available with griseofulvin ultra-micro size, PEG 400 and 8000, and PVP as polymer matrix and other excipients (colloidal silicon dioxide, lactose, magnesium stearate, methylcellulose, methylparaben and, titanium dioxide). In non-fasting groups, the peak serum level of griseofulvin was twice higher than fasting groups [134].

Recently, long-acting injectables (LAIs) of antiretroviral drugs are being explored as prospective substitutes for pill-based regimens against HIV/AIDS infection [135,136]. LAIs nano-formulations possess several advantages over conventional treatment, such as sustained drug release, increased drug bioavailability, decreased dosage and frequency of drug administration, enhanced drug stability in biological environments, decreased side effects, avoid unfavorable drug interactions, and impart specificity for pathogen-infected cells [137,138]. In 2021, two controlled/extended-release injectable NCs products under the trade name Cabenuva^®^ and Apretude^®^ were approved by the FDA against HIV-type-one (HIV-1) infection. Both products are available as a gluteal intramuscular injection in a single-dose vial. Cabenuva^®^ is a novel LAI formulation (once-monthly) with an extended-release of two drugs, cabotegravir (CAB), which is an HIV integrase nucleoside strand transfer inhibitor (INSTI), and rilpivirine, as an HIV non-nucleoside reverse transcriptase inhibitor (NNRTI) [139]. The CAB suspension was fabricated (size of 200 nm) using PEG 3350, polysorbate 20, and CAB, while the poloxamer 338 was utilized to stabilize rilpivirine suspension.

**Figure 2:**
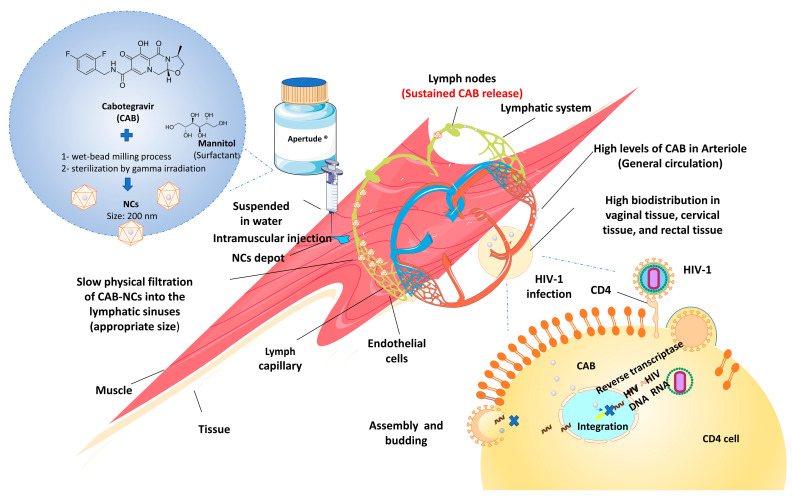
Mechanism action of Apretude (CAB-NCs) to inhibit the HIV-1 viral replication. After an intramuscular injection, CAB-NCs highly distribute in vaginal, cervical, and rectal tissue, where they bind to the active integrase site and inhibit the retroviral deoxyribonucleic acid (DNA) integration phase, which is required for the HIV replication cycle.

Apretude^®^, CAB extended-release injectable suspension, as referred by the manufacturer, is prepared by wet bead milling method and then sterilized via gamma irradiation, generalizing 200-nm particles. It is indicated for the treatment of HIV in at-risk patients or adolescents weighing more than 35 kg for pre-exposure prophylaxis (PrEP) to lower the risk of sexually acquired HIV-1 infection. The mechanism of Apretude^®^ is based on inhibiting HIV-1 viral replication (Figure 2) [140].

### 3.3. NCs for Psychosis and Other Disease Treatment

Besides the diseases mentioned above, NCs products have also been employed against various conditions such as organ rejection in renal transplantation, anorexia, psychosis, chronic pain, nausea, vomiting, etc. For instance, Rapamune^®^, immunosuppressant sirolimus NCs, was indicated to prevent organ rejection in patients aged ≥13 years receiving renal transplants. Rapamune^®^ was manufactured in 2000 by Wyeth Pharmaceuticals (Philadelphia, PA, USA) using the pearl mill method, and its oral bioavailability was found to be 21% higher than conventional formulations of sirolimus [141]. By using the same method, Megace^®^ES, an appetite stimulant NC dispersion, was developed, and it showed an increased drug dissolution rate and reduced single dose volume, thereby improving its oral bioavailability and patient compliance [142].

Invega Sustenna^®^ (2009), Invega Trinza^®^ (2015), and Invega Hafyera^®^ (2021), three NC-based products of the anti-psychotic agent (paliperidone palmitate), with extended drug release for 1 month, 3 months, and 6 months, respectively, have been approved to treat schizophrenia and schizoaffective disorder as well as to supplement mood stabilizers or antidepressants in adults. All three products with an average size of 150–200 nm were prepared via the wet media milling method using polysorbate 20 as a stabilizer and are provided in single-dose prefilled syringes with various fill volumes and strengths for intramuscular administration [140,143]. Invega Sustenna^®^ is available at a strength of 156 mg/mL. After receiving adequate treatment with Invega Sustenna for at least 4 months, patients are switched to Invega Trinza^®^, which is provided in strengths of 273 mg/0.88 mL, 410 mg/1.32 mL, 546 mg/1.75 mL, and 819 mg/2.63 mL. After completing treatment with Invega Trinza for at least one cycle, patients can begin receiving treatment with Invega Hafyera^®^ [144].

**Table 2:** Approved NC-based products.

Trade Name	Approval Year	Drug Agent	Company	Clinical Applications	Administration Route	Agency	Ref.
Gris-PEG^®^	1998	Griseofulvin	Recro Gainesville LLC (Gainesville, FL, USA)	Ringworm infections	Oral	FDA	[134]
Rapamune^®^	2000	Rapamycin/sirolimus	Wyeth (Philadelphia, PA, USA)	Immunosuppressive therapy in renal transplantation	Oral	FDA	[141]
Avinza^®^	2002	Morphine sulfate	King Pharma (Bristol, TN, USA)	Chronic pain	Oral	FDA	[145]
Ritalin LA^®^	2002	Methylphenidate hydrochloride	Novartis Novartis (Basel, Switzerland)	Attention-deficit-hyperactivity disorder	Oral	FDA	[146]
Emend^®^	2003	Aprepitant	Merck (Rahway, NJ, USA)	Chemotherapy-induced nausea and vomiting	Oral	FDA	[147]
Tricor^®^	2004	Fenofibrate	Abbott (North Chicago, IL, USA)	Hypercholesterolemia	Oral	FDA	[124]
Triglide^®^	2005	Fenofibrate	Skye Pharma (San Diego, CA, USA)	Hypercholesterolemia	Oral	FDA	[131]
Megace^®^ES	2005	Megestrol acetate	Par Pharma (Petaluma, CA, USA)	Anorexia	Oral	FDA	[142]
Naprelan^®^	2006	Naproxen sodium	Wyeth (Philadelphia, PA, USA)	Inflammation	Oral	FDA	[148]
Cesamet^®^	2009	Nabilone	Lilly (Indianapolis, IN, USA)	Nausea and vomiting	Oral	FDA	[149]
Invega Sustenna^®^	2009	Paliperidone palmitate	Janssen Pharmaceuticals Inc. (Titusville, NJ, USA)	Schizophrenia	Intramuscular	FDA	[150]
Invega Trinza^®^	2015	Paliperidone palmitate	Janssen Pharmaceuticals Inc. (Titusville, NJ, USA)	Schizophrenia	Intramuscular	FDA	[151]
Aristada Initio^®^	2018	Aripiprazole lauroxil	Alkermes Inc (Waltham, MA, USA)	Schizophrenia	Intramuscular	FDA	[152]
Invega Hafyera^®^	2021	Paliperidone palmitate	Janssen Pharmaceuticals Inc. (Titusville, NJ, USA)	Schizophrenia	Intramuscular	FDA	[144]
Cabenuva^®^	2021	Cabotegravir	Viiv Healthcare Co. (Brentford, London, UK)	HIV-1 infection	Gluteal intramuscular	FDA	[139]
Apretude^®^	2021	Cabotegravir	Viiv Healthcare Co. (Brentford, London, UK)	HIV-1 infection	Gluteal intramuscular	FDA	[140]

## 4. Polymeric Nanoparticles

Polymeric nanomedicines usually refer to nanoparticles loaded with active compounds encapsulated into the polymeric core or adsorbed onto the surface of the polymeric core. Besides lipid-based nanomedicines and NCs, polymeric nanomedicines also contribute to treating various diseases. Polymeric nanomedicines approved are listed in Table 3. The polymers used in polymeric nanomedicines are various, including natural, semi-synthetic, and synthetic polymers. From the perspective of the nanostructure, it can be divided into polymeric micelles, polymeric nanoparticles, and dendrimer-based nanoparticles [153,154].

### 4.1. Polymeric Micelles for Cancer Treatment

Polymeric micelles, formed by the self-assembly of amphiphilic block copolymers in an aqueous solution, has hydrophobic core and hydrophilic shell [155]. The hydrophobic core encapsulates drugs with low water solubility; as for the hydrophilic shell, it protects cargos and maintains the stability of the micelles [155]. The commonly used hydrophobic polymers [156,157,158] include polylactic acid (PLA), polylactide glycolide acid (PLGA), and polyamine acid (PAA), while the commonly used hydrophilic polymers are PEG, chitosan, hyaluronic acid (HA) and PVP [159,160,161]. The design of polymeric micelles aims to encapsulate active compounds to protect them from the external environment, improve their pharmacokinetic profile, and reduce the side effect [162,163]. The functionalities of the formulation depend on the properties of amphiphilic block copolymers, such as composition, surface charge, length, and molecular weight [164,165,166,167]. For example, the hydrophilic shell had a prominent place in minimizing the interaction between polymeric micelles and endogenous substances (serum proteins and complement system), avoiding unexpected leakage of drugs and quick removal by the reticuloendothelial system (RES) [165]. Xiao et al. studied the biodistribution of polymeric micelles with different surface charges [165]. The results showed extensive liver uptake of micelles with highly positive and negative charges, which may be due to the active phagocytosis of Kupffer cells in the liver. However, when the surface charge of micelles is slightly negative, the liver uptake is very low, while the tumor uptake is very high. A slightly negative charge on the surface of micelles is suggested to reduce the clearance of RES and improve blood compatibility [165]. Besides surface charge, the molecular weight of hydrophilic polymers also affects the stability and biodistribution of micelles [166,167]. Hydrophobic polymers mainly contribute to dissolving insoluble drugs and controlling drug release from the micelles [155]. The hydrophobic interaction between drugs and hydrophobic polymers is the key driving force for drug dissolving, keeping active molecules in the core and slowing its release rate [168]. Many hydrophobic polymers with a high capacity for solubilizing drugs have been synthesized [169]. There are various methods for the production of polymeric micelles, including thin film hydration [170], cosolvent evaporation [171], freeze-drying [172], dialysis [173], and supercritical fluids [174]. Particularly, thin film hydration is the most excellent among them, appropriate for large-scale production due to the fewest steps and easy removal of organic solvent [155].

So far, there have been three polymeric micelles-based nanomedicines on the market: Genexol^®^ PM, Nanoxel^®^ M, and Paclical^®^. Genexol^®^ PM is the earliest polymeric nanomedicine approved for human use in South Korea, the Philippines, India, and Vietnam in 2007. The API of Genexol^®^ PM is paclitaxel (PTX), and its indication includes metastatic breast cancer (MBC), non-small cell lung cancer (NSCLC), and ovarian cancer. The amphiphilic di-block copolymers used in Genexol^®^ PM are mPEG and poly-D,L-lactide (PDLLA) (mPEG: 2000 g/mol, PDLLA: 1750 g/mol, PDI: 1.0–1.2), having good biocompatibility and degradability [175]. The micelles are produced by thin film hydration with a size of 20–50 nm in diameter and 16.7% drug loading of PTX [175]. A preclinical study showed that the maximum tolerable dose (MTD) and median lethal dose (LD_50_) of Genexol^®^ PM were higher than those of Taxol^®^, and the biodistribution of PTX after administration of Genexol^®^ PM showed 2 to 3-fold higher levels in tissues and tumor as compared to Taxol^®^ (PTX injection, a tumor chemotherapy drug of Bristol Myers Squibb SRL, with indications of ovarian cancer and breast cancer), exhibiting a significant advantage over chemotherapy with Taxol^®^ [175]. The phase I clinical trial in South Korea recorded that the MTD for patients treated with Genexol^®^ PM was 390 mg/m^2^ while that of Taxol^®^ was 200 mg/m^2^ [176]. In the phase Ⅱ clinical trial, Genexol^®^ PM disclosed a better therapeutic effect than Taxol^®^ for treating NSCLC and MBC. In terms of safety, due to toxic excipients removal (Cremophor EL), Genexol^®^ PM has fewer side effects than Taxol^®^, increasing the compliance of patients [177].

Nanoxel^®^ M is a polymeric micelle-based preparation for cancer treatment and received approval in 2012, with docetaxel as the active drug. Similar to Genexol^®^ PM, the amphiphilic block copolymers used in Nanoxel^®^ M are mPEG and PDLLA (mPEG: 2000 g/mol, PDLLA: 1765 g/mol, PDI: 1.0~1.2) [178]. With a hydrodynamic size of 25.4 nm in diameter, the micelles are produced by the thin film hydration method. A preclinical study indicated that the IC_50_ of Nanoxel^®^ M in H-460, MCF-7, and SKOV-3 cancer cells (2.33, 1.73, and 2.19 ng/mL) were comparable to those of Taxotere^®^ (4.66, 1.83, and 3.25 ng/mL). Pharmacokinetic parameters (C_max_, AUC, t_1/2_, CL, V_ss_) in mice, rats, and beagle dogs of Nanoxel^®^ M had no significant differences with those of Taxotere^®^ (docetaxel injection, a tumor chemotherapy drug of Sanofi, with the indication of gastric cancer) [178]. These results suggested that Nanoxel^®^ M had comparable therapeutic effects with Taxotere^®^.

Paclical^®^, whose active compound is PTX, was approved for ovarian-cancer treatment in Russia in 2015 [179]. Notably, the amphiphilic surfactant XR-17 is introduced into the micellar structure of Paclical^®^. XR-17, a vitamin A analog, can form water-soluble particles with PTX. In a phase III clinical trial, Paclical^®^ demonstrated a positive risk/benefit ratio compared to a treatment based on Taxol^®^ [180]. It offers a treatment option of a higher PTX dose with a shorter infusion time without mandatory premedication [180].

In addition, many new types of polymeric micelles-based nanomedicines are in the stage of clinical trials. These polymeric micelles are primarily prepared of PEG and PAA with good biocompatibility.

### 4.2. Polymeric Nanoparticles for Cancer Treatment

Polymeric nanoparticles are solid colloidal particles composed of polymers with a size of 10~1000 nm [163]. According to nanostructure, polymeric nanoparticles can be divided into nanospheres and nanocapsules [163,181]. Nanocapsules are vesicular systems in which the drug is confined to a cavity surrounded by a unique polymer membrane, while nanospheres are matrix systems in which the drug is physically and uniformly dispersed [181]. The frequently used method for producing nanocapsules is nanoprecipitation [163]. In contrast, the methods for nanospheres are various, including nanoprecipitation, solvent evaporation, emulsification/solvent diffusion, and emulsification/reverse salting-out [163,182]. Polymeric nanoparticles share similar properties with liposomes and polymeric micelles, such as enhanced solubility, reduced toxicity, and longer circulation times [183]. In addition, compared with the two nanocarriers, polymeric nanoparticles have better stability, more uniform size distribution, and more controllable drug release through polymer matrix diffusion or erosion and particle degradation [184]. The commonly utilized polymers include natural polymers such as albumin, dextran, HA, and chitosan and synthetic polymers such as PLA, PLGA, PEG, PAA, etc. [185]. Synthetic polymers are more frequently used to produce polymeric nanoparticles due to non-required purification, easy manufacture, and sustained drug release [186].

**Figure 3:**
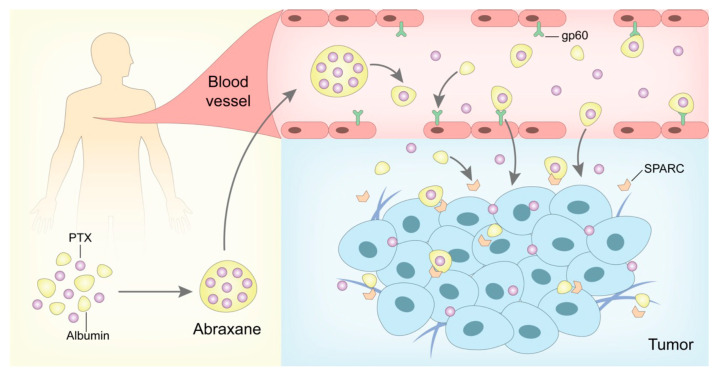
In vivo antitumor mechanism of Abraxane^®^**.** After the intravenous infusion, Abraxane^®^ could highly deliver the encapsulated PTX via albumin carrier and enhance the PTX distribution in tumor tissues mediated by gp-60 and SPARC, which has been applied in the treatment of several kinds of malignant melanoma.

Polymeric nanoparticles can be loaded with various active compounds, such as antitumor drugs, siRNA, proteins, and contrast agents [183]. So far, there has been only one commercialized polymeric nanoparticle: Abraxane^®^. FDA approved Abraxane^®^ in 2005 for the treatment of pancreatic cancer and MBC. Abraxane^®^ is an albumin-bound, 130-nm particle formulation of PTX [187] (Figure 3). In a clinical trial, Abraxane^®^ not only maintains the antitumor effect of PTX but also eliminates the toxicity related to Cremophor^®^ EL in Taxol^®^ [188]. The pharmacokinetic study showed that the PTX clearance rate and tumor distribution capacity of Abraxane^®^ were higher than Taxol^®^ due to the ligand-receptor targeting effect by the active albumin transport pathway [188]. In addition, Abraxane^®^ was approved to treat NSCLC in 2012, and its phase III clinical trial for the treatment of malignant melanoma is undergoing [189,190].

### 4.3. Dendrimer-Based Nanoparticles against Infection

Dendrimers are branched polymers with repeating units, which are spherical and symmetrical in an aqueous solution [191]. The most widely used dendrimers involve polyamide amine (PAMAM), polyether-copolyester (PEPE), polyimide (PPI), and gallic acid-triethylene glycol (GATG) [192]. These dendrimers have unique structural and physicochemical properties, including (1) spherical, highly branched structure; (2) mono-dispersity, low viscosity; (3) various drug loading methods and high payload capacity; (4) controllable nanometer size; (5) the terminal amino group is convenient for functional modification [193,194]. The distinctive molecular structure of dendrimers leads to a dense periphery and a loose core of the particles, which can be used to carry various drugs such as small molecular drugs, nucleic acids, and diagnostic agents [195,196,197,198]. Dendrimers are rich in amino groups on the surface and can also be loaded with drugs through chemical coupling [199,200]. Therefore, dendrimer-based nanoparticles can deliver various drugs to different lesions.

So far, there has been only one marketed dendrimer nanomedicine: Viva-Gel^®^. Viva-Gel^®^ was approved in 2006 for preventing HIV and herpes simplex virus (HSV) indication. SPL7013, the active ingredient of Viva-Gel^®^, is a dendrimer with a specifically designed polyanionic surface, which enables SPL7013 to attach to viruses, blocking viral attachment or adsorption to cells, thereby preventing infection [201]. Numerous preclinical studies showed that SPL7013 effectively protected human cells from HIV and HSV infection, indicating a therapeutic potential for SPL7013 [202,203,204]. In a clinical trial, 36 healthy women received either VivaGel™ containing 0.5–3.0% *w*/*w* SPL7013 or a placebo (the base Carbopol^®^ formulation without SPL7013) once daily intravaginally for a week [205]. All SPL7013 concentrations of VivaGel™ exhibited good safety and tolerance as a placebo [205]. Besides, SPL7013 was not absorbed into the systemic circulation, verifying its safety [205]. Further clinical studies showed that VivaGel™ containing >0.5% SPL7013 could inhibit more than 70% of HIV-1 and HSV-2, with activity maintained for at least 3 h post-dose [206].

**Table 3:** Approved polymeric nanoparticle-based products.

Trade Name	Approval Year	Drug Agent	Company	Clinical Applications	Administration Route	Agency	Ref.
Genexol^®^ PM	2007	PTX	Samyang Pharmaceuticals (Seoul, Republic of Korea)	MBC, NSCLC, and ovarian cancer	Intravenous	MFDS, PDH, CDSCO and DAV	[175]
Nanoxel^®^ M	2012	Docetaxel	Samyang Pharmaceuticals (Seoul, Republic of Korea)	MBC, NSCLC, and ovarian cancer	Intravenous	MFDS	[178]
Paclical^®^	2015	PTX	Oasmia Pharmaceuticals (Uppsala, Sweden)	Ovarian cancer	Intravenous	RFMPH	[180]
Abraxane^®^	2005	PTX	Abraxis Bioscience, (Los Angeles, CA, USA)	Pancreatic cancer and MBC	Intravenous	FDA	[187]

MFDS, Ministry of Food and Drug Safety (Status elevated from Republic of Korea Food and Drug Administration); PDH, Philippines: Department of Health; CDSCO, Central Drug Standards Control Organization of India; DAV, Drug Administration of Vietnam; RFMPH, Russian Federation: Ministry of Public Health.

## 5. Other Nanomedicines for Disease Treatment

In addition to the marketed nanomedicines previously described, there are currently a variety of other nanomedicines approved for the clinical treatment of various diseases (Table 4). With the rapid development of NDDSs via advanced synthetic or natural biological materials, various novel nanomedicines, different from conventional nanomedicines, have been developed into antibody-drug conjugates, cell-derived vehicles, viral vectors, inorganic nanoparticles, protein-based nanoparticles [15,207]. These nanomedicines have been broadly applied to treat or diagnose specific indications, including cancer, infectious diseases, inflammation, blood disorders, immunological diseases, CVDs, nervous system diseases, mental diseases, endocrine and metabolic diseases, etc. [208]. Meanwhile, many new nanomedicines or some previous nanomedicines applied for different indications are still in clinical trials (phase I/II/III), which need to be validated with extensive clinical data before their final transition [209,210,211].

Ontak^®^ (Denileukin diftitox, Seragen Inc., Teynampet, India) is the first genetically constructed fusion protein and is considered a recombinant molecule that combines a targeting mechanism with a cytocidal moiety [212]. Possessing a unique mechanism of action, Ontak^®^ can direct and lead the cytocidal action of diphtheria toxin toward all the cells overexpressing the interleukin-2 (IL-2) receptor. This medicine could be effectively internalized into IL-2 receptor-bearing cells by endocytosis, and the IL-2 gene inside is genetically fused to the enzymatically active and translocating domains of diphtheria toxin, inducing apoptosis. So far, Ontak^®^ has been proven for clinical application in various diseases, such as Hodgkin’s disease, rheumatoid arthritis, psoriasis, B-cell non-Hodgkin’s lymphoma, cutaneous T-cell lymphoma (CTCL), and HIV infection.

Furthermore, nanomedicine can also be used for the topical treatment of eye diseases. Dry eye disease (DED) is caused by various genetic and/or environmental factors, characterized by excessive tear evaporation or tear deficiency, the pathogenesis of which is lacrimal gland inflammation and hyperpermeability of the tear film and has become a common chronic disease [213,214]. Cyclosporine is widely used in treating DED due to its anti-inflammatory activity, but its hydrophobic properties greatly limit its ocular administration [215]. Restasis^®^, a cyclosporin nanoemulsion, can significantly improve cyclosporine’s solubility and prolong the ocular surface’s retention time by emulsions [216]. A large number of clinical application results confirmed that, after being administrated with Restasis^®^, the tear secretion was significantly increased, and the therapeutic effect in patients with systemic disease is better than in patients without systemic disease.

**Table 4:** Summary of other launched nanomedicine for treating multiple diseases.

Trade Name	Approval Year	Formulation Type	Drug Agent	Company	Clinical Applications	Administration Route	Agency	Ref.
Ontak^®^	1999	Protein-based formulation	Denileukin diftitox	Eisai (Norcross, GA, USA)	Cutaneous T-cell lymphoma therapy	Intravenous	FDA	[212]
Oncaspar^®^	1994	Pegylated enzyme	L-asparaginase	Enzon Pharmaceuticals (Cranford, NJ, USA); Baxter BioScience (Deerfield, IL, USA)	Acute lymphocytic leukemia	Intravenous	FDA	[217]
Restasis^®^	2002	Nanoemulsions	Cyclosporin	Allergan (Lansing, MI, USA)	Severe keratitis in dry eye patient	Topical	FDA	[216]
Feraheme™	2009	Semi-synthetic iron oxide nanoparticles	Iron oxide particles	AMAG Pharmaceuticals (Waltham, MA, USA)	Anemia related to chronic kidney disease (CKD)	Intravenous	FDA	[218]
Injectafer^®^	2013	Iron nanoparticles	Polynuclear iron (III) oxyhydroxide iron particles	For Int. (Waltham, MA, USA)	Iron deficiency anemia	Oral	FDA	[219]
Monofer^®^	2010	Iron nanoparticles	Iron molecule with unbranched carbohydrate iron particles	Pharmacosmos (Rorvangsvej, Holbæk, Denmark)	Iron deficiency anemia	Oral	EMA/FDA	[220]
Mircera^®^	2007	Polymer-protein conjugate	Methoxy polyethylene glycol-epoetin beta	Hoffman-LaRoche (Basel, Switzerland)	CKD associated anemia	Intravenous	FDA	[221]
Adynovate^®^	2015	Polymer-protein conjugate	Recombinant anti-hemophilic factor VIII	Baxalta (Montgomery, AL, USA)	Hemophilia A	Intravenous	FDA	[222]
Neulasta^®^	2002	Polymer-protein conjugate	Recombinant human granulocyte-colony stimulating factor (G-CSF)	Amgen (Thousand Oaks, CA, USA)	Febrile neutropenia	Intravenous	FDA	[223]
Pegasys^®^	2002	Pegylated nanoparticles	Interferon alfa-2a	Genentech biotechnology (San Francisco, AL, USA)	Hepatitis B and C therapy	Intravenous	FDA	[224]
Pegintron^®^	2001	Pegylated nanoparticles	Interferon alfa-2b	Merck (Rahway, NJ, USA)	Hepatitis C	Intravenous	FDA	[225]
Copaxone^®^	1996	Polypeptide colloidal formulation	Glatiramer acetate	Teva Pharmaceuticals (Marietta, GA, USA)	Relapsing or remitting type of multiple sclerosis	Intravenous	FDA	[226]
Estrasorb^®^	2003	Emulsion	Estradiol	Novavax (Lutherville Timonium, MD, USA)	Estrogen therapy	Topical	FDA	[227]
Nanocoll^®^	1995	Albumin-based radiopharmaceutical nanocolloid	Albumin and stannous	GE Healthcare (Raleigh, NC, USA)	Breast cancer and also melanoma	Intravenous	FDA	[228]
Nanocis^®^	2000	Radiopharmaceutical colloid	Chloride dehydrates Radiopharmaceutical colloid	CIS Bio (Berlin, Germany)	As inflammation scintigraphy, bone marrow scintigraphy, and by cutaneous route for lymphatic scintigraphy	Intravenous	FDA	[229]

FDA: Food and Drug Administration; EMA: European Medicines Agency.

## 6. Conclusions and Perspectives

Nanomedicine has obtained significant advancement, evident by the vast number of publications and commercialized products (about 90 approved nanomedicines). Significantly, the co-delivery nanomedicine Vyxeos (for AML, 2017) and siRNA-loaded LNPs, Onpattro (for treating hATTR polyneuropathy), were approved. Since 2005, the FDA has streamlined the filing process for nanomedicines to facilitate their development [230,231]. The process enabled dozens of liposomes, NCs, and LNPs to be marketed in the last decade, including two products for COVID-19 treatment, Comirnaty and mRNA-1273. Liposomes and NCs are the two most successful nanomedicine, accounting for over 60% of the marketed products. The two NDDSs have significant advantages in drug-loading capacity compared to other nanocarriers; meanwhile, liposomes often demonstrate well-acceptable safety and robust ability to protect the drugs from degradation due to their closed structure. As a result, the three factors are vital to nanomedicine translation.

Given over 90 nanomedicines have been commercialized, the transition proportion is still low compared to the massive number of publications. Over 50,000 research articles on nanomedicine in 2018–2022, only nine products entered the market [17,232]. The factors that hinder the clinical application are complex, such as the modest EPR effect in cancer patients, unclear in vivo fate, and toxicity. The EPR effect is often demonstrated in animal tumor models and, in contrast, is humble in patients with tumors. The EPR differences between animal models and humans are an unignored factor limiting transition [233]. Safety is the most important aspect of drug development. For the accumulation improvement in the lesion site, it is generally inclined to reduce the particle size of nanomedicine, but the smaller size also increases accumulation in the spleen and liver, resulting in safety concerns [234]. Third, the in vivo fate of nanomedicine is poorly demonstrated because of the absence of effective strategies to explore the metabolism. Fortunately, the in vivo fate is being concerned. Increasing novel techniques are emerging for the fate exploration of nanoparticles, e.g., radioactive tracing and fluorescence bioimaging using environment-responsive fluorescent probes based on aggregation-caused quenching and aggregation-induced emission and förster resonance energy transfer [235,236,237]. Additionally, researchers found the protein corona (PC) in the nanoparticles in the blood dramatically affects the in vivo fate of nanomedicine, such as target ability, biodistribution, stability, and toxicity [238]. A deep understanding of the PC on the nanocarriers’ biological fate could, in turn, facilitate the rational design of nanoformulation and application. Additionally, nanoformulation’s quality control, i.e., diameter and size distribution, morphology, surface charge, drug loading, and release profile, remains challenging. The involvement of quality by design (QBD) to combine process and product development, GMP-compliant production conditions, and multidisciplinary effort may help the translation [239]. The microfluidic technique, especially, represents a promising approach for the quality management of nanomedicine because it can offer several advantages for manufacturing, including process control and feedback for constantly managing production and procedure control, outstanding design flexibility, parameter setting, etc. [239].

Nonetheless, the difficulty in translation would not impede the rapid development of nanomedicine due to its considerable benefits in improving the delivery of biopharmaceuticals with high specificity and potency. Always, nanoencapsulation enables side-effect reduction for small molecular drugs. For instance, DOX liposomes exhibited similar anti-tumor activity with the free drug but significantly decreased cardiotoxicity. Whereas, for biological drugs, nanotechnology is potent to enhance their treatment efficacy and reduce side effects by elevating their stability, barrier-crossing capacity, and intracellular delivery. E.g., nucleic acid drugs display huge potential to treat various major human diseases; yet, their application problem was not addressed over decades due to the drawbacks, including large size, hydrophilicity, instability, negative charge, and poor membrane penetration. The emergence of LNPs allows their clinical use to be achieved. The first siRNA drug, Onpattro™, approved in 2018, represents a breakthrough for genetic medicine from concept to clinical use and opens an era for the clinical use of biopharmaceuticals. Afterward, increasing nucleic acid drugs were commercialized using the LNP technology, such as two mRNA vaccines, mRNA-1273 and BNT162b2. As a result, we can anticipate that nanotechnology will continuously make a considerable impact on biopharmaceutical development and increasing biologics-related nanomedicine will be marketed in the future.

## Data Availability

Data sharing not applicable. No new data were created or analyzed in this study. Data sharing is not applicable to this article.
